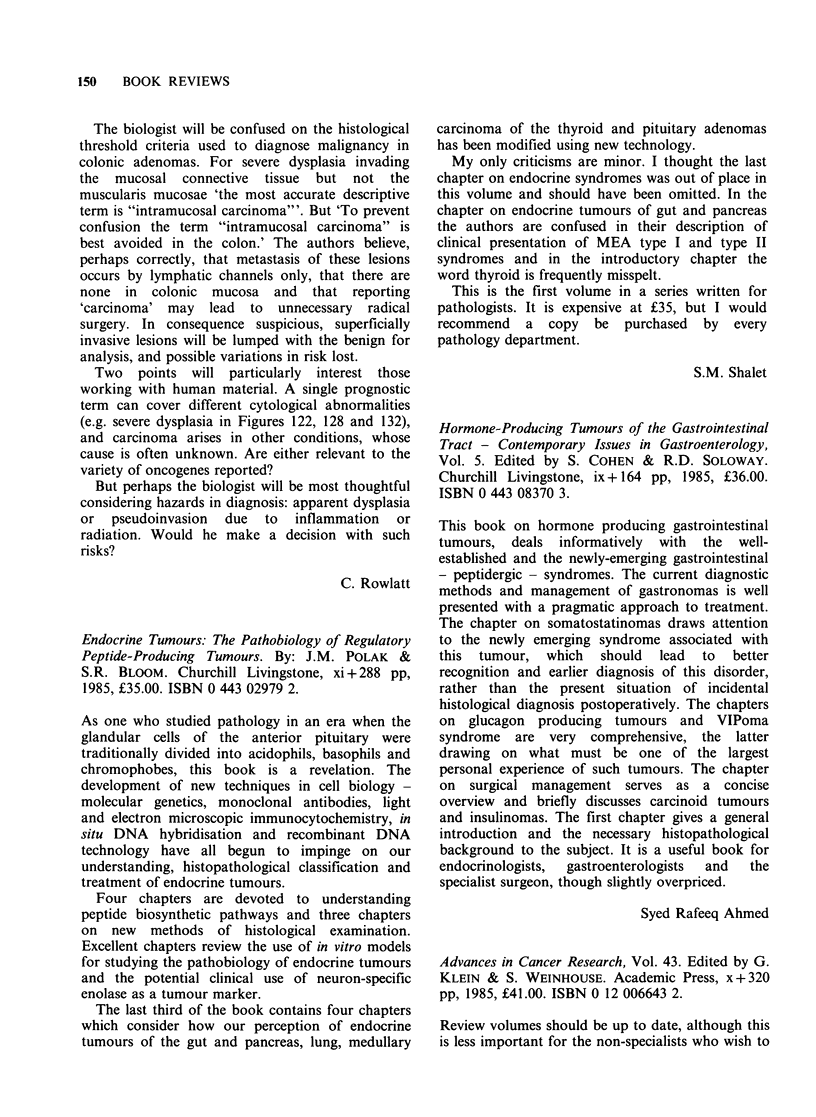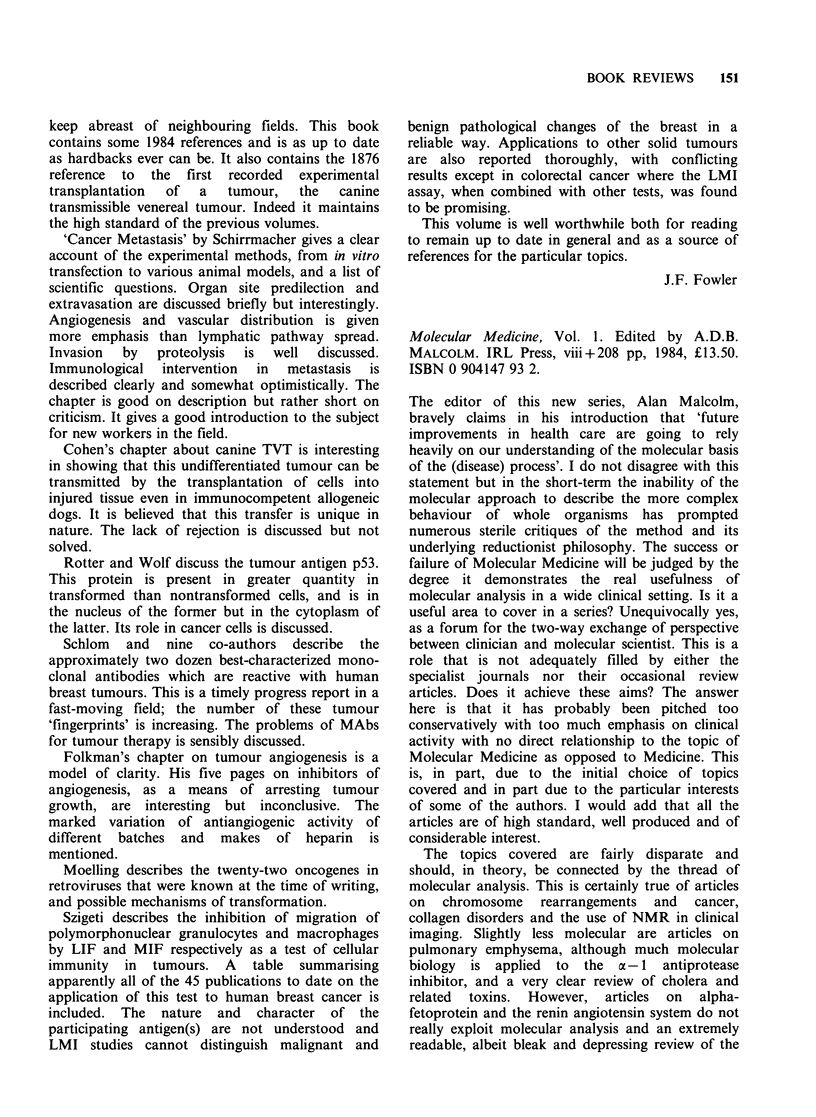# Advances in Cancer Research

**Published:** 1986-01

**Authors:** J.F. Fowler


*Advances in Cancer Research,* Vol. 43. Edited by G. Klein & S. Weinhouse. Academic Press, x+320 pp, 1985, £41.00. ISBN 0 12 006643 2.

Review volumes should be up to date, although this is less important for the non-specialists who wish to keep abreast of neighbouring fields. This book contains some 1984 references and is as up to date as hardbacks ever can be. It also contains the 1876 reference to the first recorded experimental transplantation of a tumour, the canine transmissible venereal tumour. Indeed it maintains the high standard of the previous volumes.

'Cancer Metastasis' by Schirrmacher gives a clear account of the experimental methods, from *in vitro* transfection to various animal models, and a list of scientific questions. Organ site predilection and extravasation are discussed briefly but interestingly. Angiogenesis and vascular distribution is given more emphasis than lymphatic pathway spread. Invasion by proteolysis is well discussed. Immunological intervention in metastasis is described clearly and somewhat optimistically. The chapter is good on description but rather short on criticism. It gives a good introduction to the subject for new workers in the field.

Cohen's chapter about canine TVT is interesting in showing that this undifferentiated tumour can be transmitted by the transplantation of cells into injured tissue even in immunocompetent allogeneic dogs. It is believed that this transfer is unique in nature. The lack of rejection is discussed but not solved.

Rotter and Wolf discuss the tumour antigen p53. This protein is present in greater quantity in transformed than nontransformed cells, and is in the nucleus of the former but in the cytoplasm of the latter. Its role in cancer cells is discussed.

Schlom and nine co-authors describe the approximately two dozen best-characterized monoclonal antibodies which are reactive with human breast tumours. This is a timely progress report in a fast-moving field; the number of these tumour 'fingerprints' is increasing. The problems of MAbs for tumour therapy is sensibly discussed.

Folkman's chapter on tumour angiogenesis is a model of clarity. His five pages on inhibitors of angiogenesis, as a means of arresting tumour growth, are interesting but inconclusive. The marked variation of antiangiogenic activity of different batches and makes of heparin is mentioned.

Moelling describes the twenty-two oncogenes in retroviruses that were known at the time of writing, and possible mechanisms of transformation.

Szigeti describes the inhibition of migration of polymorphonuclear granulocytes and macrophages by LIF and MIF respectively as a test of cellular immunity in tumours. A table summarising apparently all of the 45 publications to date on the application of this test to human breast cancer is included. The nature and character of the participating antigen(s) are not understood and LMI studies cannot distinguish malignant and benign pathological changes of the breast in a reliable way. Applications to other solid tumours are also reported thoroughly, with conflicting results except in colorectal cancer where the LMI assay, when combined with other tests, was found to be promising.

This volume is well worthwhile both for reading to remain up to date in general and as a source of references for the particular topics.

J.F. Fowler